# Correction: Reactions of nitroxides 16. First nitroxides containing tellurium atom

**DOI:** 10.1039/d2ra90044k

**Published:** 2022-05-04

**Authors:** Jerzy Zakrzewski, Bogumiła Huras, Anna Kiełczewska, Maria Krawczyk

**Affiliations:** Institute of Industrial Organic Chemistry Annopol 6 03-236 Warsaw Poland zakrzewski@ipo.waw.pl

## Abstract

Correction for ‘Reactions of nitroxides 16. First nitroxides containing tellurium atom’ by Jerzy Zakrzewski *et al.*, *RSC Adv.*, 2016, **6**, 98829–98834, https://doi.org/10.1039/c6ra15880c.

The authors regret that incorrect text concerning names of specific chemical compounds were included in the original article.

In the subheading “3-(Phenyltellanyl)ethylamine **3a**, 3-(phenyltellanyl)propylamine **3b** (according to a known procedure^20^ with some modifications)” on page 98831, ‘3-(Phenyltellanyl)ethylamine **3a**’ should read as ‘2-(Phenyltellanyl)ethylamine **3a**’.

In the subheading “3-(Phenyltellanyl)ethylamine **3a** (*M* = 248.6)” on page 98831, ‘3-(Phenyltellanyl)ethylamine **3a**’ should read as ‘2-(Phenyltellanyl)ethylamine **3a**’.

In the subheading “3-(Phenyltellanyl)ethyl isothiocyanate **4a**, and 3-(phenyltellanyl)propyl isothiocyanate **4b**. General procedure” on page 98831, ‘3-(Phenyltellanyl)ethyl isothiocyanate **4a**’ should read as ‘2-(Phenyltellanyl)ethyl isothiocyanate **4a**’.

Following the subheading “Tellurium nitroxides **6a–6f**, general procedure” on page 98832, in the sentence reading “2-(Phenyltellanyl)ethyl isothiocyanate (**4a**, 0.111 g, 0.382 mmol) or 2-(phenyltellanyl)propyl isothiocyanate”, ‘2-(phenyltellanyl)propyl isothiocyanate’ should read as ‘3-(phenyltellanyl)propyl isothiocyanate’.

The Royal Society of Chemistry apologises for these errors and any consequent inconvenience to authors and readers.

## Supplementary Material

